# Association between smoking and COVID-19 severity: A multicentre retrospective observational study

**DOI:** 10.1097/MD.0000000000029438

**Published:** 2022-07-22

**Authors:** Yue He, Yangai He, Qinghui Hu, Sheng Yang, Jun Li, Yuan Liu, Jun Hu

**Affiliations:** a Department of Infectious Diseases, the First Affiliated Hospital of Nanjing Medical University, Nanjing, China; b Department of Orthopedics, the First Affiliated Hospital of Nanjing Medical University, Nanjing, China; c Department of Biostatistics, Nanjing Medical University, Nanjing, China.

**Keywords:** coronavirus disease 2019, disease severity, nicotine, random forest, severe acute respiratory syndrome coronavirus 2, smoking

## Abstract

The relationship between smoking and coronavirus disease 2019 (COVID-19) severity remains unclear. This study aimed to investigate the effect of smoking status (current smoking and a smoking history) on the clinical severity of COVID-19. Data of all enrolled 588 patients, who were referred to 25 hospitals in Jiangsu province between January 10, 2020 and March 14, 2020, were retrospectively reviewed. Univariate and multivariate regression, random forest algorithms, and additive interaction were used to estimate the importance of selective predictor variables in the relationship between smoking and COVID-19 severity. In the univariate analysis, the proportion of patients with a current smoking status in the severe group was significantly higher than that in the non-severe group. In the multivariate analysis, current smoking remained a risk factor for severe COVID-19. Data from the interaction analysis showed a strong interaction between the number of comorbidities in patients with COVID-19 and smoking. However, no significant interaction was found between smoking and specific comorbidities, such as hypertension, diabetes, etc. In the random forest model, smoking history was ranked sixth in mean decrease accuracy. Active smoking may be significantly associated with an enhanced risk of COVID-19 progression towards severe disease. However, additional prospective studies are needed to clarify the complex relationship between smoking and COVID-19 severity.

## 1. Introduction

Coronavirus disease 2019 (COVID-19), caused by severe acute respiratory syndrome coronavirus 2 (SARS-CoV-2), was declared a global public health emergency by the World Health Organization in January, 2020.^[1]^ Whole genome sequencing and pathogenic nucleic acid analysis confirmed that the virus was the seventh member of the coronavirus family that can infect humans.^[2]^ As of March 2021, the number of persons with COVID-19 worldwide was approximately 115 million, with more than 2.5 million deaths.^[3]^ COVID-19 has several clinical manifestations. The main presenting symptoms are fever and respiratory symptoms, such as cough, sputum production, and shortness of breath. Some patients present with extrapulmonary symptoms, such as diarrhoea, cardiac function injury, and liver and kidney function injury. Some patients with severe disease may present with acute respiratory distress syndrome, multiple organ dysfunction syndromes, and even death.^[4]^

SARS-CoV-2 is a β coronavirus, which is 88% identical to two bat-derived severe acute respiratory syndrome (SARS)-like coronaviruses, bat-SL-CoVZC45 and bat-SL-CoVZXC21, at the whole genome level. However, the S receptor-binding domain structure of SARS-CoV-2 is similar to that of SARS-CoV, despite the amino acid variation at some key residue points.^[5]^ The receptor of SARS-CoV-2 and SARS-CoV is angiotensin-converting enzyme 2 (ACE2).^[6]^ They invade cells through endosomal or non-endosomal pathways with the help of different host cell proteases, which have a structural basis for direct injury of extrapulmonary organs or tissues. Because of the cell specificity of ACE2 and host cell protease, the affinity of SARS-CoV-2 to different organs varies.^[7,8]^

Smoking seriously damages human health and is the main risk factor for respiratory and cardiovascular diseases.^[9]^ ACE and ACE2 are important components of the renin-angiotensin system. ACE is an enzyme that catalyses the conversion of angiotensin (Ang) I to Ang II, which exerts a strong vasoconstrictive effect. Ang II favours vasoconstriction, cellular proliferation, inflammation, and fibrilization when it binds to the Ang II Type 1 receptor. Previous studies have suggested that long-term smoking changes renin-angiotensin system homeostasis by up-regulating the detrimental ACE/Ang II/Ang II type 1 receptor axis and down-regulating the compensatory ACE2/Ang-(1-7)/Mas receptor axis, thus favouring the development of cardiopulmonary diseases.^[9,10]^ Thus, it would be expected that a large proportion of patients with COVID-19 are smokers. Chakladar et al found that smoking-mediated upregulation of the androgen pathway leads to increased SARS-CoV-2 susceptibility.^[11]^ Umnuaypornlert et al conducted a meta-analysis and found that smoking, whether current smoking or former smoking, significantly increases the risk of COVID-19 severity and death.^[12]^

In contrast, a systematic review and meta-analyses found an unexpectedly low prevalence of current smoking among hospitalised patients with COVID-19.^[13,14]^ A low prevalence of current smokers among hospitalised patients with COVID-19 has been reported in several studies.^[15,16]^ However, the relationship between smoking and COVID-19 severity remains unclear. To address this important clinical question, this study aimed to evaluate the effect of smoking status (current smoking and former smoking) on the clinical severity of COVID-19, which may provide additional evidence for active and effective interventions and treatment measures for early-disease stages and for reducing the mortality rate of patients with severe disease.

## 2. Materials and Methods

### 2.1. Study design and participants

A total of 588 patients were referred to 25 hospitals in Jiangsu province between January 10, 2020 and March 14, 2020; they were enrolled, and their data were retrospectively and consecutively analysed. According to the government's arrangements, all tertiary hospitals provide treatment for patients with COVID-19, diagnosed using the World Health Organization interim guidance^[17,18]^ and the guidelines of COVID-19 diagnosis and treatment trial (5th edition) of the National Health Commission of the People's Republic of China.^[18,19]^ This study was performed in accordance with the Helsinki Declaration and was approved by the Ethics Committee of the First Affiliated Hospital of Nanjing Medical University. Written informed consent was obtained from participants or their families for data collection. Based on disease severity, the patients were divided into four groups: mild, moderate, severe, and critically ill. The criteria for this clinical classification can be found in previous studies.^[20,21]^

### 2.2. Data collection and study definitions

The variables of interest included (1) participants’ general information: age, sex, smoking history, comorbidities (such as chronic obstructive pulmonary disease, hypertension, diabetes, cardiovascular disease, cerebrovascular disease, hepatitis B, cancer, chronic kidney disease, and immunodeficiency disease), therapeutic drugs, respiratory support, and disease outcome; (2) patients’ main clinical symptoms and signs; (3) results of laboratory tests performed within 48 hours of admission to the hospital or intensive care unit: blood routine examination, level of C-reactive protein (CRP), procalcitonin, lactate dehydrogenase, aspartate aminotransferase (AST), alanine aminotransferase, total bilirubin, creatine kinase, creatinine, D-dimer, erythrocyte sedimentation rate (ESR), and finger pulse oxygen saturation (SaO_2_); and (4) imaging findings such as chest computed tomography findings. Data were obtained from the electronic medical records and initially evaluated by trained physicians. Full recovery and discharge, disease regression from critical/severe to non-severe disease status, positive to negative polymerase chain reaction, and maintenance of non-severe status were considered as disease improvement or favourable clinical outcome.

### 2.3. Smoking history

Smoking was quantified as pack-years (number of cigarettes smoked per day multiplied by the number of years of smoking). For example, smoking one pack a day for 10 years was defined as 10 pack-years. Any patient with a smoking quantity above 10 pack-years was considered as having a significant smoking history. Based on the patients’ smoking history, the study population was divided into two groups: the ≥10 pack-years group (16 patients), which included patients whose current smoking was ≥10 pack-years, and the <10 pack-years group (572 patients), which included non-smokers, former smokers, and patients whose current smoking was <10 pack-years.

### 2.4. Statistical analysis

Continuous variables are expressed as medians and interquartile ranges or simple ranges, as appropriate. Categorical variables are summarised as counts and percentages. For continuous variables, the Student *t* test or Mann–Whitney *U* test was used for data analysis, while the chi-square test or Fisher exact test was used to analyse categorical variables. No imputation was made for missing data. All the statistics in this study are descriptive because the patient cohort was not derived from random selection. Odds ratios and 95% confidence intervals were calculated using univariate and multivariate logistic regression models. The random Forest package in R software was used to perform a random forest classification of the data.^[22–24]^ To assess the relationship between independent and dependent variables, COVID-19 severity was considered as the dependent variable and clinical characteristics as the independent variables. The number of classification trees was set at 1000. Each time, 588 patients were sampled randomly with replacement to construct the classification tree. In each split, entry was set as the square root of the number of total variables to sample variables randomly as candidates. The variables were assessed and ranked by measuring the effect of perturbing them on Mean Decrease Accuracy (MDA) and Mean Decrease Gini (MDG). Comorbidity–smoking behaviour interaction analysis was performed using a logistic regression model. All analyses were implemented with R 3.12 software (R Foundation for Statistical Computing, Beijing, China; http://www.Rproject.org).^[24]^ Two-sided *P*-values <.05 were considered statistically significant.

## 3. Results

### 3.1. Demographic and clinical characteristics

All the 588 patients had positive reverse transcriptase polymerase chain reaction tests. By the end of the study, all the patients had recovered and were discharged. The severe group consisted of 46 patients with severe or critical disease, while the non-severe group consisted of 542 patients with mild or moderate disease. The demographic and clinical characteristics of the patients in the two groups are shown in Table 1. There was no significant difference in sex between the two groups. The median age of the patients was 46 (range, 33–56) years. Most of the patients in the severe group were above 65 years (34.8%, 16/46), while most of the patients in the non-severe group were within the 15 to 49 years age range (58.9%, 319/542). On admission, cough (87.0%, 40/46) and fever (43.5%, 20/46) were the most common symptoms in the severe group, while cough (64.0%, 347/542) and fever (30.8%, 167/542) were the most common in the non-severe group. Hypertension (16.3%, 96/588) and diabetes (7.8%, 46/588) were the most common comorbidities in the overall patient cohort, severe group (34.8%, 16/46; 26.1%, 12/46), and non-severe group (14.8%, 80/542; 6.3%, 34/542).

**Table 1:** Demographics and baseline characteristics of patients with COVID-19.

	Severe cases (n = 46)	Nonsevere cases (n = 542)	*P* ‡
Age			
Median (IQR), y	56.5 (48.0–68.5)	44.0 (32.0–55.0)	
Distribution no./total no. (%)			
0–14 y	0/46 (0.0)	19/542 (3.5)	
15–49 y	15/46 (32.6)	319/542 (58.9)	
50–64 y	15/46 (32.6)	153/542 (28.2)	
=65 y	16/46 (34.8)	51/542 (9.4)	
Female sex no./total no. (%)	21/46 (45.7)	266/542 (49.1)	
Smoking history no./total no. (%)			.056
Never smoked	40/46 (87.0)	509/542 (93.9)	.111
Former smoker	0/46 (0.0)	9/542 (1.7)	>.99
Current smoker	6/46 (13.0)	24/542 (4.4)	.023
Median length of hospital stay (IQR) days	21.5 (16.0–27.0)	16.0 (12.0–20.0)	
Fever on admission			
Patients no./total no. (%)	20/46 (43.5)	167/542 (30.8)	.108
Median temperature (IQR) °C	36.9 (36.5–37.7)	36.8 (36.5–37.4)	.600
Distribution of temperature no./total no. (%)			.443
	33/46 (71.7)	410/542 (75.6)	
37.5–38.0 °C	6/46 (13.0)	84/542 (15.5)	
38.1–39.0 °C	6/46 (13.0)	42/542 (7.7)	
>39.0 °C	1/46 (2.2)	6/542 (1.1)	
Symptoms no. (%)			
Conjunctival congestion	1 (2.2)	4 (0.7)	.336
Nasal congestion	0 (0.0)	25 (4.6)	.248
Dizziness and headache	6 (13.0)	59 (10.9)	.839
Cough	40 (87.0)	347 (64.0)	.003
Sore throat	2 (4.3)	53 (9.8)	.297
Fatigue	15 (32.6)	150 (27.7)	.586
Shortness of breath	16 (34.8)	40 (7.4)	
Nausea or vomiting	6 (13.0)	26 (4.8)	.031
Diarrhea	2 (4.3)	33 (6.1)	>.99
Myalgia or arthralgia	4 (8.7)	67 (12.4)	.619
Chills	6 (13.0)	71 (13.1)	>.99
Chest distress	14 (30.4)	73 (13.5)	.004
Chest pain	1 (2.2)	5 (0.9)	.388
Inappetence	15 (32.6)	83 (15.3)	.005
Signs no. (%)			
Throat congestion	14 (30.4)	191 (35.2)	.620
Tonsil swelling	0 (0.0)	11 (2.0)	1.000
Enlargement of lymph nodes	0 (0.0)	0 (0.0)	
Rash	0 (0.0)	0 (0.0)	
Coexisting disorders no. (%)			
Chronic obstructive pulmonary disease	2 (4.3)	6 (1.1)	.124
Hypertension	16 (34.8)	80 (14.8)	.001
Diabetes	12 (26.1)	34 (6.3)	
Cardiovascular disease	2 (4.3)	16 (3)	.644
Cerebrovascular disease	3 (6.5)	7 (1.3)	.037
Hepatitis B infection*	1 (2.2)	7 (1.3)	.481
Cancer†	3 (6.5)	6 (1.1)	.027
Chronic renal disease	0 (0)	4 (0.7)	>.99
Immunodeficiency	2 (4.3)	4 (0.7)	.073
Laboratory findings			
SaO2 < 95% no./total no. (%)	11/28 (39.29)	15/180 (8.33)	
White-cell count			
Median (IQR) ×109/L	4.90 (3.77–7.80)	4.75 (3.82–5.95)	.378
Distribution no./total no. (%)			
>10 × 109/L	8/44 (18.2)	6/455 (1.3)	
9/L	15/44 (34.1)	137/455 (30.1)	.707
Lymphocyte count			
Median (IQR) ×109/L	0.71 (0.61–1.08)	1.32 (0.95–1.66)	
Distribution no./total no. (%)			
9/L	26/45 (57.8)	63/440 (14.3)	
Platelet count			
Median (IQR) ×109/L	157 (126–209)	178 (146–221)	.074
Distribution no./total no. (%)			
9/L	17/43 (39.5)	109/402 (27.1)	.124
Median hemoglobin (IQR) g/L	136 (124–148)	139 (128–152)	.209
Distribution of other findings no./total no. (%)			
C-reactive protein =10 mg/L	22/36 (61.1)	149/369 (40.4)	.026
Procalcitonin =0.5 ng/mL	4/33 (12.1)	16/315 (5.1)	.109
Lactate dehydrogenase =250 U/L	26/36 (72.2)	155/374 (41.4)	.001
Aspartate aminotransferase >40 U/L	12/30 (40.0)	45/322 (14.0)	.001
Alanine aminotransferase >40 U/L	12/38 (31.6)	80/384 (20.8)	.185
Total bilirubin >17.1 µmol/L	5/39 (12.8)	73/400 (18.2)	.530
Creatine kinase =200 U/L	5/28 (17.9)	24/325 (7.4)	.067
Creatinine =133 µmol/L	1/41 (2.4)	2/383 (0.5)	.264
D-dimer =0.5 mg/L	15/40 (37.5)	85/373 (22.8)	.061
Erythrocyte sedimentation rate (IQR) mm/h	50.0 (14.0–70.0)	18.0 (8.0–32.0)	
Minerals			
Median sodium (IQR) mmol/L	138.7 (136.0–141.0)	139.0 (136.0–141.0)	.036
Median potassium (IQR) mmol/L	3.8 (3.5–4.1)	3.8 (3.5–4.1)	.272
CD4+ T cells counts (IQR) cells/µL	363 (234–489)	534.0 (384.0–765.8)	.009
CD4+/CD8+ ratio (IQR)	1.70 (1.32–2.83)	1.62 (1.19–2.14)	.554
Radiologic findings			
CT evidence of pneumonia no./total no. (%)			.425
Typical signs of viral infection (Ground-glass opacity or patchy shadowing)	26/46 (56.5)	310/542 (57.2)	
Atypical signs (Stripe shadowing)	18/46 (39.1)	177/542 (32.7)	
Normal	2/46 (4.3)	55/542 (10.1)	

CI = confidence interval, Covid-19 = coronavirus disease 2019, IQR = interquartile range.

* The presence of hepatitis B infection was defined as a positive result on testing for hepatitis B surface antigen with or without elevated levels of alanine or aspartate aminotransferase.

† Included in this category is any type of cancer.

‡*P* values comparing severe cases and nonsevere cases are from χ^2^ test, Fisher's exact test, or unpaired 2-sided Student *t* test, or Mann–Whitney *U* test.

### 3.2. Univariate and multivariate analysis results

The univariate analysis showed that there were significant differences in age, current smoking status, length of hospital stay, cough, shortness of breath, nausea or vomiting, chest distress, inappetence, comorbidities such as hypertension, diabetes, cerebrovascular disease, and cancer, SaO_2_ <95%, white blood cell count >10.0 × 10^9^/L, lymphocyte count <0.8 × 10^9^/L, CRP level ≥10 mg/L, LDH level ≥250 U/L, AST level >40 U/L, ESR, sodium level, and CD4^+^ T cells counts between the two groups (*P* < .05, Table 1). In the multivariate regression analysis, age, current smoking status, length of hospital stay, cough, cerebrovascular disease, SaO_2_ <95%, and lymphocyte count <0.8 × 10^9^/L were risk factors for severe COVID-19 (*P* < .05, Table 2). Some factors such as shortness of breath, nausea or vomiting, chest distress, inappetence, comorbidities such as hypertension, diabetes, and cancer, white blood cell count >10.0 × 10^9^/L, CRP level ≥10 mg/L, LDH level ≥250 U/L, AST level >40 U/L, ESR, sodium level, and CD4^+^ T cell counts were not significant factors in the multivariate regression analysis.

**Table 2:** Multivariate analysis of risk factors related to the incidence of the severe cases.

Variable	OR (95% CI)	*P*
Age	1.04 (1.01, 1.08)	.015
Current smoker	4.28 (1.04, 15.72)	.033
Length of hospital stay	1.07 (1.01, 1.12)	.015
Cough	6.18 (2.14, 22.37)	.002
Shortness of breath	1.96 (0.57, 6.44)	.273
Nausea or vomiting	3.93 (0.91, 14.84)	.052
Chest distress	1.23 (0.35, 3.86)	.730
Inappetence	1.49 (0.59, 3.59)	.383
Hypertension	1.52 (0.56, 3.92)	.397
Diabetes	1.57 (0.50, 4.57)	.421
Cerebrovascular disease	7.64 (0.87, 54.08)	.046
Cancer	1.22 (0.07, 13.50)	.882
[Table_Body]SaO_2_	0.15 (0.04, 0.56)	.004
Lymphocyte count ^9^/L	6.17 (2.67, 14.45)	[Table_Body]
C-reactive protein =10 mg/L	0.81 (0.34, 1.84)	.621
Aspartate aminotransferase >40 U/L	2.49 (0.84, 6.74)	.082
Median sodium	0.70 (0.27, 1.66)	.431
CD4^+^ T cells counts	1.00 (0.99, 1.00)	.092

### 3.3. Relationship between smoking and COVID-19 severity

In the univariate analysis, the proportion of patients with a current smoking status in the severe group (13.0%, 6/46) was significantly higher than that in the non-severe group (4.4%, 24/542). Current smoking status remained a risk factor for COVID-19 severity in the multivariate analysis. (*P* < 05). The differences in the demographic and clinical characteristics between the ≥10 pack-years and <10 pack-years group are shown in Table 3. There were significant differences in age, sex, diabetes, cerebrovascular disease, median haemoglobin level, and CD4^+^ T cell counts between the two groups.

**Table 3:** Comparison between characteristics of COVID-19 patients currently smoking 10+ cigarette pack years versus Others†.

	10+ Pack years (n = 16)	Others* (n = 572)	*P* †
Age			
Median (IQR) y	51.0 (49.75–58.0))	45.0 (32.0–56.0))	.006
Distribution no./total no. (%)			.011
0–14 y	0/16 (0.0)	19/572 (3.3)	
15–49 y	4/16 (25.0)	330/572 (57.7)	
50–64 y	10/16 (62.5)	158/572 (27.6)	
=65 y	2/16 (12.5)	65/572 (11.4)	
Female sex no./total no. (%)	1 (6.25)	286/572 (50.0)	.001
Median length of hospital stay (IQR) days	16.0 (14.5–19.25)	16.0 (13.0–21.0)	.987
Fever on admission			
Patients no./total no. (%)	5/16 (31.2)	182/572 (31.8)	>.99
Median temperature (IQR) °C	36.8 (36.67–38.0)	36.8 (36.5–37.4)	.529
Distribution of temperature no./total no. (%)			.453
	11/16 (68.8)	432/572 (75.5)	
37.5–38.0 °C	2/16 (12.5)	88/572 (15.4)	
38.1–39.0 °C	3/16 (18.8)	45/572 (7.9)	
>39.0 °C	0/16 (0.0)	7/572 (1.2)	
Symptoms no. (%)			
Conjunctival congestion	0 (0.0)	5 (0.9)	>.99
Nasal congestion	1 (6.2)	24 (4.2)	.506
Dizziness and headache	4 (25.0)	61 (10.7)	.089
Cough	9 (56.2)	378 (66.1)	.581
Sore throat	0 (0.0)	55 (9.6)	.385
Fatigue	5 (31.2)	160 (28.0)	.781
Shortness of breath	3 (18.8)	53 (9.3)	.189
Nausea or vomiting	1 (6.2)	31 (5.4)	.597
Diarrhea	1 (6.2)	34 (5.9)	>.99
Myalgia or arthralgia	4 (25.0)	67 (11.7)	.115
Chills	1 (6.2)	76 (13.3)	.708
Chest distress	1 (6.2)	86 (15.0)	.488
Chest pain	0 (0.0)	6 (1.0)	>.99
Inappetence	4 (25.0)	94 (16.4)	.321
Signs no. (%)			
Throat congestion	3 (18.8)	202 (35.3)	.269
Tonsil swelling	0 (0.0)	11 (1.9)	>.99
Enlargement of lymph nodes	0 (0.0)	0 (0.0)	
Rash	0 (0.0)	0 (0.0)	
Coexisting disorders no. (%)			
Chronic obstructive pulmonary disease	0 (0.0)	8 (1.4)	>.99
Hypertension	5 (31.2)	91 (15.9)	.158
Diabetes	5 (31.2)	41 (7.2)	.005
Cardiovascular disease	1 (6.2)	17 (3.0)	.396
Cerebrovascular disease	2 (12.5)	8 (1.4)	.028
Hepatitis B infection	0 (0.0)	8 (1.4)	>.99
Cancer	0 (0.0)	9 (1.6)	>.99
Chronic renal disease	0 (0.0)	4 (0.7)	>.99
Immunodeficiency	1 (6.2)	5 (0.9)	.153
Laboratory findings			
SaO_2_ < 95% no./total no. (%)	2/6 (33.33)	24/202 (11.88)	.165
White-cell count			
Median (IQR) ×10^9^/L	5.450 (4.375–6.950)	4.750 (3.810–5.935)	.074
Distribution no./total no. (%)			
>10 × 10^9^/L	2/16 (12.5)	12/483 (2.5)	.070
^9^/L	2/16 (12.5)	150/483 (31.1)	.166
Lymphocyte count			
Median (IQR) ×10^9^/L	1.0300 (0.8175–1.4600)	1.250 (0.890–1.630)	.341
Distribution no./total no. (%)			
^9^/L	4/16 (25.0)	85/469 (18.1)	.510
Platelet count			
Median (IQR) ×10^9^/L	159.0 (132.5–225.0)	177.0 (144.0–220.0)	.403
Distribution no./total no. (%)			
^9^/L	6/15 (40.0)	120/430 (27.9)	.381
Median hemoglobin (IQR) g/L	144 (140.8–151)	138 (127–152)	.044
Distribution of other findings no./total no. (%)			
C-reactive protein =10 mg/L	10/15 (66.7)	161/390 (41.3)	.092
Procalcitonin =0.5 ng/mL	0/9 (0.0)	20/339 (5.9)	>.99
Lactate dehydrogenase =250 U/L	6/15 (40.0)	175/395 (44.3)	.948
Aspartate aminotransferase >40 U/L	3/16 (18.8)	54/336 (16.1)	.731
Alanine aminotransferase >40 U/L	3/14 (21.4)	89/408 (21.8)	>.99
Total bilirubin >17.1 µmol/L	5/15 (33.3)	73/424 (17.2)	.158
Creatine kinase =200 U/L	1/10 (10.0)	28/343 (8.2)	.581
Creatinine =133 µmol/L	1/15 (6.7)	2/409 (0.5)	.103
D-dimer =0.5 mg/L	6/13 (46.2)	94/400 (23.5)	.093
Erythrocyte sedimentation rate (IQR) mm/h	49.5 (3–61)	18 (9–33)	.474
Minerals			
Median sodium (IQR) mmol/L	139.0 (134.5–141.9)	138.7 (136.0–141.0)	.985
Median potassium (IQR) mmol/L	3.9 (3.6–4.0)	3.8 (3.5–4.1)	.635
CD4^+^ T cells counts (IQR) cells/uL	723.5 (570.5–833.0)	481.0 (365.5–728.0)	.043
CD4^+^/CD8^+^ ratio (IQR)	1.840 (1.545–2.067)	1.600 (1.200–2.285)	.695
Radiologic findings			
CT evidence of pneumonia no./total no. (%)			.929
Typical signs of viral infection (Ground-glass opacity or patchy shadowing)	9/16 (56.2)	327/572 (57.2)	
Atypical signs (Stripe shadowing)	6/16 (37.5)	189/572 (33.0)	
Normal	1/16 (6.2)	56/572 (9.8)	

CI = confidence interval, Covid-19 = coronavirus disease 2019, IQR = interquartile range.

* Others were defined as patients who have never smoked, who have quit smoking and who are currently smoking but have not smoked more than 10+ Pack-years.

† *P* values comparing severe cases and nonsevere cases are from χ^2^ test, Fisher's exact test, or unpaired 2-sided Student *t* test, or Mann–Whitney *U* test.

Epidemiological studies show an interaction among factors when the combined effect of more than two risk factors on a disease is different from the sum of their independent effects. Biological interaction has attracted much attention due to its practicality in biological and clinical settings, as it relies heavily on statistical interactions.^[22]^ The evaluation of statistical interactions is mainly based on multiplicative and additive interactions, with additive interaction having a greater biological and public health significance.^[23]^ Through the interaction analysis, we found that only the number of comorbidities had an interaction with smoking (*P* = .043), with estimates of interaction (odds ratio, 0.58; 95% confidence interval: 0.31–0.95) (Table 4). There was no interaction between smoking and chronic obstructive pulmonary disease, hypertension, diabetes, cardiovascular disease, cerebrovascular disease, hepatitis B, cancer, chronic kidney disease, and tuberculosis (*P* > .05).

**Table 4:** Characteristics of COVID-19 patients–smoking interaction analysis.

Interaction	OR (95% CI)	*P*
Coexisting disorder		
COPD × Smoking	1.98 × 10^-7^ (0, 1.33 × 10^49^)	.988
Hypertension × Smoking	0.34 (6.31 × 10^-2^, 1.11)	.114
Diabetes ×	0.75 (0.22, 2.31)	.615
Cardiovascular disease × Smoking	2.64 × 10^-6^ (0, 6.38 × 10^34^)	.986
Cerebrovascular disease Smoking	2.93 × 10^-4^ (0, 1.18 × 10^31^)	.987
Cancer Smoking	4.94 × 10^-4^ (0, 4.26 × 10^35^)	.986
Chronic renal disease × Smoking	0.59 (5.22 × 10^-14^, 6.61 × 10^12^)	>.99
Tuberculosis Smoking	7.86 × 10^-4^ (0, 6.34 × 10^29^)	.987
Combined		
Number of coexisting disorders × Smoking	0.58 (0.31, 0.95)	.043

**Figure 1.**
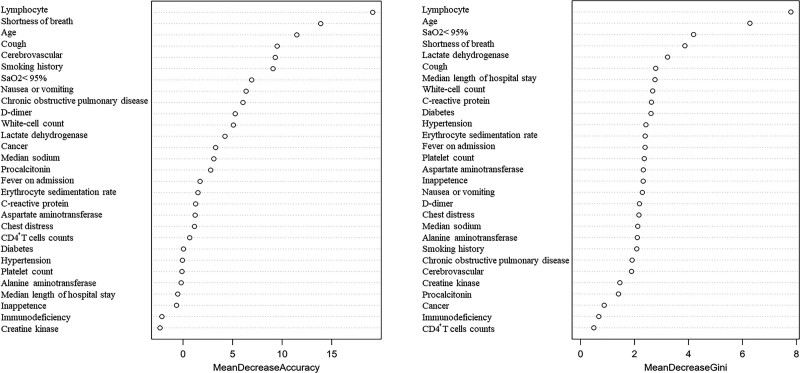
The ranking chart of the two accuracy indicators. Variable selection by a random forest using mean decreases in accuracy and the Gini index, according to which the importance score of each variable was calculated.

The random forest algorithm can be used to analyse nonlinear, collinear, and interactive data effectively. As one of the classical algorithms of machine learning, the random forest algorithm has a high accuracy in risk prediction and diagnosis of diseases. It is currently widely used in molecular and genetic fields and other medical fields.^[24,25]^ In MDA, the greater the decrease in the accuracy after permutation of the variable, the more important the predictor is. MDG) is the sum of all decreases in Gini impurity. MDG values show the importance of the variable in COVID-19 severity prediction. The top three important predictors in the MDA analysis were lymphocyte count, shortness of breath, and age. The top three variables in the MDG analysis were lymphocyte count, age, and SaO_2_ <95%. Smoking history was ranked sixth in the MDA analysis and twenty second in the MDG analysis (Fig. 1).

## 4. Discussion

In the univariate analysis in this study, the proportion of patients with a current smoking status in the severe group was significantly higher than that in non-severe group. Current smoking status still remained a risk factor for severe COVID-19 in the multivariate analysis. The interaction analysis showed that there was a strong interaction between the number of comorbidities in patients with COVID-19 and smoking. However, there was no significant interaction between smoking and specific comorbidities, such as hypertension, diabetes, etc. In the random forest model, smoking history was ranked sixth in MDA.

The tobacco industry has created millions of jobs around the world and provided a large amount of tax revenue to the government. Simultaneously, 50% of tobacco consumers die from cigarette smoke, which causes heavy losses to the healthcare system. During the COVID-19 pandemic, smoking and the risk of acute respiratory infections have attracted a lot of interest again.^[26,27]^ Smith et al demonstrated that ACE2 is expressed in a subset of secretory cells in the respiratory tract. Chronic smoke exposure triggers an increase in the population of these cells and a concomitant increase in ACE2 expression. ACE2 expression is responsive to inflammatory signalling and can be upregulated by viral infections or interferon treatment. They suggested that SARS-CoV-2 infections could create positive feedback loops that increase ACE2 levels and facilitate viral dissemination.^[28]^ These mechanisms may partially explain why smokers are particularly susceptible to severe SARS-CoV-2 infections. Findings from Leung et al suggested that quitting smoking can reduce the probability of COVID-19 progression to severe disease. In the study, patients who were smokers or who had chronic obstructive pulmonary disease had higher ACE2 levels, increasing the probability of viral entry into the host cells and infection. They also found that ex-smokers and never-smokers had similar ACE2 levels. These findings support the fact that immediate quitting of smoking is optimal.^[29]^ Arunima also found that smoking causes more severe SARS-CoV-2 infection by blocking the activity of the immune system messenger proteins, interferons, at least in part. Interferons play a crucial role in the body's early immune response, triggering infected cells to produce proteins that attack the virus, getting extra support from the immune system, and alerting uninfected cells to prepare to fight the virus. Smoking can prevent an effective interferon-based response to the SARS-CoV-2 virus.^[30]^

A French clinical observational study reported that current smokers had a lower susceptibility to SARS-CoV-2 infection, although the disease was severe once they got infected.^[31]^ Some meta-analyses also reported a low prevalence of current smoking among hospitalized patients with COVID-19.^[14]^ The mechanism of action of nicotine may explain the paradox of the relationship between smoking and COVID-19. When COVID-19 gets severe, excessive lung inflammation may occur due to a virus-activated “cytokine storm.”. The cholinergic anti-inflammatory pathway that modulates the inflammatory response during systemic inflammation has been demonstrated. In addition, α7-nicotinic acetylcholine receptor is essential in attenuating the inflammatory response.^[32]^ Nicotine is the main active substance in tobacco. It has been reported that nicotine, an agonist, plays an anti-inflammatory role in mice with acute lung injury.^[33]^ Some studies suggested that nicotine may represent a potential therapeutic target for the improvement of cytokine storms and attenuation of dysregulated inflammatory responses of patients with COVID-19.^[32,34]^ Perhaps this complex biological mechanism of nicotine can explain different relationships between smoking and COVID-19 severity in epidemiological studies.

It is believed that older patients with chronic diseases, such as diabetes, cardiovascular diseases, and hypertension are susceptible to respiratory failure and may have a poorer outcome.^[15,35]^ In our study, in the univariate analysis, the proportion of patients with hypertension, diabetes, cerebrovascular disease, or cancer in the severe group was significantly higher than that in the non-severe group. Hypertension, diabetes, cerebrovascular disease, and cancer remained risk factors for severe COVID-19 in the multivariate analysis. Interestingly, there were statistically significant differences in diabetes and cerebrovascular disease between the patients who were current smokers with a smoking quantity of ≥10 pack-years and the other patients. The internal regulatory mechanism of diabetes, cerebrovascular disease, and smoking needs further research.

This study has three main limitations that must be acknowledged. First, due to the study's retrospective nature and the limited number of patients, our conclusions need to be further verified in prospective studies with large sample sizes. Second, data on prognosis was unavailable at the time of the analysis, and a longer follow-up time would have provided more detailed information on the potential risk factors that could interfere with clinical outcomes. Third, the study was focused on patients with obvious clinical symptoms who went to the hospital for treatment; thus, asymptomatic patients who might have been super-spreaders or patients with mild symptoms may have been missed. Fourth, smoking is a behavioural change in a person, and COVID has infected all throughout the world irrespective of their habits. Our retrospective analysis may only assume an association between smoking status and risk of severe COVID-19, and not a cause-and-effect relation.^[36]^ Considering the descriptive nature of the current reports with no control group, and other factors, our findings should be considered as hypothesis-generating indicating the need for further studies.^[37]^ Fifth, patients with lung cancer are more susceptible and more likely to develop more severe COVID-19 disease after SARS-CoV-2 infection.^[38,39]^ So further study by collecting more patients with COVID-19 complicated by lung cancer to investigate the relationship between lung cancer, smoking and COVID-19 may be required.

## 5. Conclusions

In conclusion, the results of this multicentre retrospective observational study of Chinese patients suggest that active smoking may have a significant association with a greater risk of COVID-19 progressing towards severe disease. Physicians and public health professionals should urgently take effective preventive measures to fight against tobacco use by assisting smokers to quit smoking successfully, and thereby curb the COVID-19 pandemic. However, we also insist that additional prospective studies are needed to clarify the complex relationship between smoking and COVID-19.

## Author contributions

Conceptualization: Jun Hu and Yuan Liu.

Formal analysis: Jun Hu and Sheng Yang.

Funding acquisition: Jun Hu and Yuan Liu.

Investigation: Yue He and Yangai He.

Methodology: Jun Hu and Sheng Yang.

Project administration: Jun Hu and Yuan Liu.

Resources: Jun Li.

Software: Sheng Yang.

Supervision: Jun Hu, Yuan Liu, and Jun Li.

Validation: Jun Hu and Qinghui Hu.

Visualization: Jun Hu and Sheng Yang.

Writing – original draft preparation: Y. H. and Yangai He.

Writing – review & editing: Jun Hu and Qinghui Hu.

Conceptualization: Jun Hu, Yuan Liu

Formal analysis: Jun Hu, Sheng Yang

Funding acquisition: Jun Hu, Yuan Liu

Investigation: Yangai He, Yue He

Methodology: Jun Hu, Sheng Yang

Project administration: Jun Hu, Yuan Liu

Resources: Jun Li

Software: Sheng Yang

Supervision: Jun Hu, Jun Li, Yuan Liu

Validation: Jun Hu, Qinghui Hu

Visualization: Jun Hu, Sheng Yang

Writing – original draft: Yangai He, Yue He

Writing – review & editing: Jun Hu, Qinghui Hu
